# Real-Time Continuous Glucose Monitoring Reduces the Duration of Hypoglycemia Episodes: A Randomized Trial in Very Low Birth Weight Neonates

**DOI:** 10.1371/journal.pone.0116255

**Published:** 2015-01-15

**Authors:** Florence Uettwiller, Aude Chemin, Elisabeth Bonnemaison, Géraldine Favrais, Elie Saliba, François Labarthe

**Affiliations:** 1 Médecine Pédiatrique, CHRU de Tours, Université François Rabelais, Tours, France; 2 Réanimation Pédiatrique et Néonatologie, CHRU de Tours, Université François Rabelais, Tours, France; 3 Inserm U930, Université François Rabelais, Tours, France; 4 Inserm U1069, Université François Rabelais, Tours, France; Université Paris Descartes; AP-HP, Groupe Hospitalier Cochin-Saint-Vincent-de-Paul, FRANCE

## Abstract

**Objectives:**

Hypoglycemia is frequent in very low birth weight (VLBW) neonates and compromises their neurological outcome. The aim of this study was to compare real-time continuous glucose monitoring system (RT-CGMS) to standard methods by intermittent capillary blood glucose testing in detecting and managing hypoglycemia.

**Study design:**

Forty-eight VLBW neonates were enrolled in this prospective study. During their 3 first days of life, their glucose level was monitored either by RT-CGMS (CGM-group), or by intermittent capillary glucose testing (IGM-group) associated with a blind-CGMS to detect retrospectively missed hypoglycemia. Outcomes were the number and duration of hypoglycemic (≤50mg/dl) episodes per patient detected by CGMS.

**Results:**

Forty-three monitorings were analyzed (IGM n = 21, CGM n = 22), with a median recording time of 72 hours. In the IGM group, blind-CGMS revealed a significantly higher number of hypoglycemia episodes than capillary blood glucose testing (1.2±0.4 vs 0.4±0.2 episode/patient, p<0.01). In the CGM-group, the use of RT-CGMS made it possible (i) to detect the same number of hypoglycemia episodes as blind-CGMS (1.2±0.4 episode/patient), (ii) to adapt the glucose supply in neonates with hypoglycemia (increased supply during days 1 and 2), and (iii) to significantly reduce the duration of hypoglycemia episodes per patient (CGM 44[10–140] min versus IGM 95[15–520] min, p<0.05). Furthermore, it reduced the number of blood samples (CGM 16.9±1.0 vs IGM 21.9±1.0 blood sample/patient, p<0.001).

**Conclusion:**

RT-CGMS played a beneficial role in managing hypoglycemia in VLBW neonates by adjusting the carbohydrate supply to the individual needs and by reducing the duration of hypoglycemia episodes. The clinical significance of the biological differences observed in our study need to be explored.

## Introduction

Very low birth weight (VLBW) neonates are at a high risk of neonatal hypoglycemia, presumably due to their limited reserves of glycogen and fat, mainly constituted during the third quarter of pregnancy [1]. Moreover, preterm VLBW neonates are not able to use alternative substrates, such as ketone bodies, for energy production in case of hypoglycemia [2]. In experimental models, hypoglycemia induces cerebral energy failure, with increased excitotoxicity and oxidative stress responsible for protein nitration and lipid peroxidation, recently confirmed in premature newborns [3, 4, 5, 6]. Recurrent and prolonged episodes of hypoglycemia are then associated with serious brain damage and poor neurodevelopmental outcome [7, 8, 9, 10]. In a resource limited country, Khan *et al.* reported that hypoglycemia increases by more than 24 fold the risk ratio of neurodevelopmental delay at 6 months of age of premature neonates (p<0,05) [11]. Neuroimaging studies in newborn suffering from neonatal hypoglycemia show diffuse cerebral cortical and subcortical white matter damage, predominantly in the parietal and occipital lobes, which patterns of damage could result from regional hypoperfusion and excitatory toxicity with cell-type-specific injury [12, 13]. However, the definition of “significant hypoglycemia”, i.e. the level of glucose concentration and duration required to cause neurological damage, remains confused [14]. This glucose level probably differs depending on term and birth weight. Furthermore, severe hypoglycemia episodes may occur without concomitant clinical symptoms [7, 15]. Therefore, systematic blood glucose monitoring and rapid treatment even for mild hypoglycemia are recommended for VLBW infants during the neonatal period [16]. This is classically achieved by repetitive capillary blood glucose testing [17]. Recently, continuous glucose monitoring systems (CGMS) have been developed for the management of diabetes mellitus, initially with a retrospective analysis of the glucose measurements and currently with a real-time readout which can be used to manage glucose level [18]. These devices have also been adapted to investigate glucose homeostasis during the neonatal period and some studies have demonstrated that their use was safe and reliable for neonates [19, 20, 21]. However, until now, the glucose monitoring devices have not been used in real-time readout or to manage neonatal hypoglycemia. We conducted a prospective study using real-time CGMS (RT-CGMS) to detect hypoglycemia in VLBW neonates (Continuous glucose monitoring group or CGM-group). In the control group, glucose level was monitored by intermittent capillary blood glucose testing and blind-CGMS that was analyzed retrospectively to detect missed hypoglycemia (Intermittent glucose monitoring group IGM-group). We hypothesized that CGMS detects more hypoglycemia episodes than capillary blood glucose testing and that its use in real time (RT-CGMS) could reduce the number and duration of hypoglycemia episodes.

## Patients and Method

The protocol for this trial and supporting CONSORT checklist are available as supporting information; see S1 CONSORT Checklist and S1 Protocol.

### Ethics Statement

Approval was obtained for this study by the local Ethic Committee (Comité de Protection des Personnes CPP Tours Ouest-1). Informed consent was signed by the parents before inclusion. The present study has been registered with the ClinicalTrials.gov registry (N°NCT01942239; under the name RTCGMS) retrospectively, because registration in a WHO-approved registry was not systematic at the time of the trial design.

### Patients

Very low birth weight (VLBW) preterm infants (birth weight ≤1500g) who were admitted before 24 hours of life to the Neonatal Intensive Care Unit of the University Hospital of Tours were eligible for this prospective study. Exclusion criteria were a serious congenital abnormality, a skin condition that contraindicated continuous glucose monitoring, a transfer to another hospital during the first days of life or an absence of parental agreement.. The study was planned to cover a period of 12 months because of limitations in the availability of the CGMS devices. The number of neonates reaching inclusion criteria during one year was 48 patients. After enrollment, patients were randomized with stratification according to their birth weight (≤1000g, and 1001–1500g). The random allocation sequence was automatically generated by the statistical software of the University of Tours, with 8 patients per block. Two series (one per birth weight category) of numbered and sealed envelopes were created, containing a note with the device to be used for each patient. Each envelope was opened in order after the enrollment of each patient. Patients were enrolled by the principal investigator (FU) between November 2008 and November 2009.

### Data Collection

All data were collected in the Neonatal Intensive Care Unit of the University Hospital of Tours. All the stored data (RT- and blind-CGMS) were then secondarily transferred to an online securized database and analyzed retrospectively with an access restricted to the principal investigator.

### Glucose Monitoring

Glucose level was monitored either by RT-CGMS (CGM-group, Minimed Guardian^®^ RT, Medtronic^®^, Nothridge, USA), or by intermittent capillary glucose testing (IGM-group) associated with a blind-CGMS (Guardian Clinical^®^, Medtronic^®,^ Nothridge, USA kindly supplied by Medtronic^®^) to detect retrospectively missed hypoglycemia. Both devices were used to manage glucose levels in the interstitial tissue. They are constituted of a sensor connected to a transmitter. The sensor is a flexible platinum electrode coated with glucose oxidase that converts glucose concentration into an electric signal every 10 seconds. The electric signal is transmitted by radio frequency to the external monitor that averages it every 5 minutes and converts the signal into glucose level, providing 288 interstitial glucose concentration data points per day stored in the device. Briefly, the sensor was inserted into the subcutaneous tissue of the child’s thigh after analgesia by oral glucose (30% dextrose solution) and local anesthesia with lidocaïne (Emla) according to gestational age. After withdrawal of the needle, the sensor was connected to the transmitter and then fixed with a transparent adhesive. The sensor and the transmitter were similar in both groups. These glucose levels were used in real-time readout only with the RT-CGMS and analyzed retrospectively with the blind-CGMS in the second group. These devices require twice-daily blood samplings for calibration and have reliable threshold between 40 and 400 mg/dl. For our study, hypoglycemia was defined as interstitial glucose level ≤50 mg/dl. Consecutive or <30 min data points ≤50 mg/dl were defined as a single episode of low glucose concentration. The mean number of hypoglycemic episodes per patient has been calculated depending on the total number of patients per group. The duration of each hypoglycemic episode has been defined as the duration from the first CGMS data point ≤50 mg/dl to the last data point ≤50 mg/dl in the same hypoglycemic episode. Median [min-max] values of duration per patient were calculated concerning only patients with hypoglycemic episodes, excluding patients who did not experience hypoglycemia.

Except for the calibration protocol, which was similar in both groups, IGM- and CGM-groups had different protocols for glucose level monitoring. In the IGM-group, capillary blood glucose testing was performed every 4 hours using glucose reagent strips Optium H^®^ and the glucose meter Optium Xceed^®^ (Abbott®, Abbott Diabetes Care Inc., Alameda, USA). In the CGM group, glucose values ≤60 mg/dl were notified by an alarm; they were then controlled by capillary blood testing. In both groups, when the capillary blood glucose level was between 50 and 60 mg/dl, the intravenous glucose supply was increased by 1 g/kg/day and the glucose level was controlled 2 hours later. Hypoglycemia episodes (≤50 mg/dl) were treated by an intravenous bolus of 10% dextrose (3 ml/kg) followed by an increase of continuous glucose supply (+2 g/kg/day), and was controlled 30 to 60 min later. All patients received enteral and intravenous fluids and caloric supplies according to the clinical guidelines of the Neonatal Intensive Care Unit of the University Hospital of Tours. Patient’s characteristics were recorded in the files. Maternal-fetal infection was determined by the pediatrician in charge of the child from clinical, biological and bacterial arguments, according to conventional guidelines. The primary outcome was to compare the number of hypoglycemic episodes per patient detected either by intermittent capillary blood glucose testing or by CGMS in this population. The secondary outcomes were to compare the number of hypoglycemia episodes per patient detected by RT- or blind-CGMS, the duration of these episodes, the number of blood samples per patient for glucose determination, and the daily carbohydrate intake and caloric supplies in both groups.

### Statistical Analysis

Collected data included the characteristics of the patients, the number of glucose capillary samples, the retrospective analysis of CGMS data and modifications of enteral and intravenous fluids and caloric supplies. Post-randomization excluded patients are reported in the characteristics of the patients but were excluded from further analyses. Results are expressed as means ± SE, median [min-max], or frequencies (percentages) when appropriate. Statistical analyses were performed using software SAS version 9.1.3 (Cary, NC, USA). Given that most of the data did not follow a normal distribution and that the sample size was small, non-parametric tests (Mann-Withney) were used to compare continuous variables, except for the number of blood samples for capillary blood glucose testing that was analyzed using Student t-test. Categorical variables were analyzed using Fisher test. The accuracy of glucose level estimated by the CGMS was compared to the corresponding capillary blood glucose value by a linear regression test. The number of hypoglycemic episodes/patient detected either by CGMS or by capillary blood glucose testing was compared by a Wilcoxon matched pairs test. The mean daily caloric and carbohydrate supplies were analyzed by Two-way repeated measures ANOVA. A p-value <0.05 was considered statistically significant.

## Results

### Patient Characteristics

**Figure 1:**
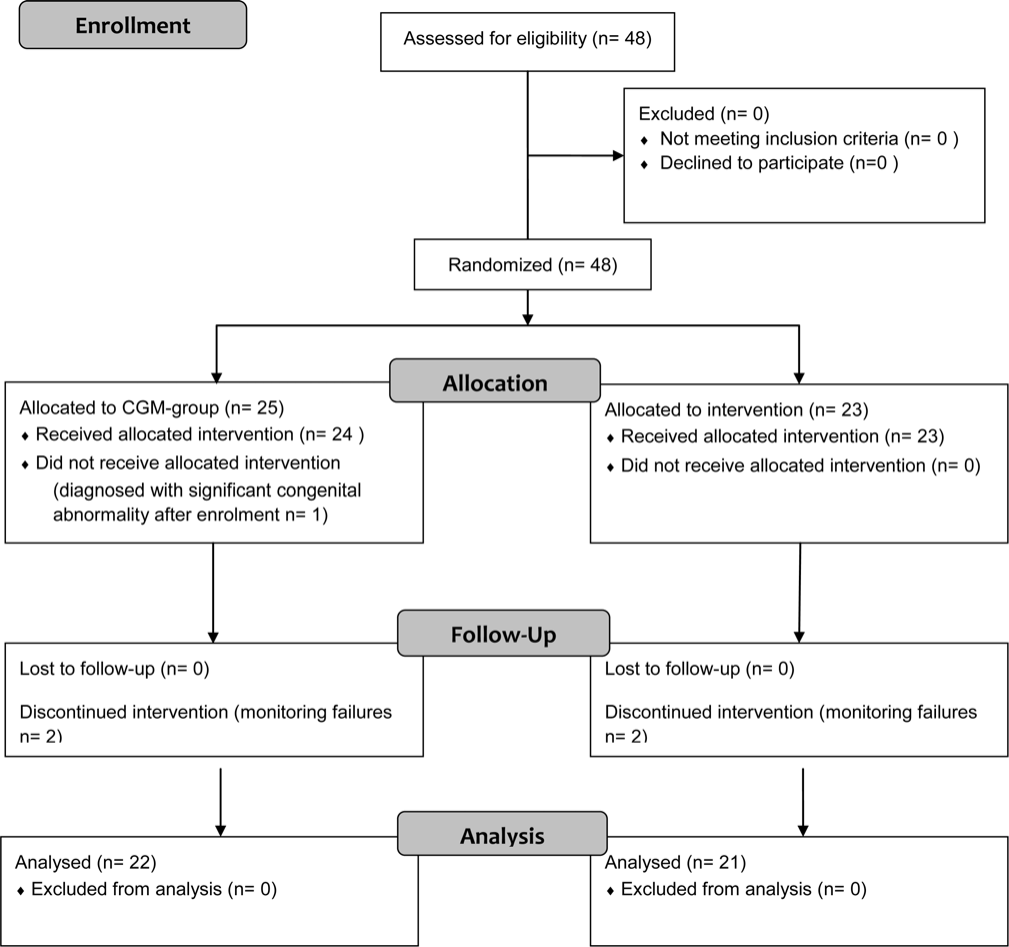
Flow chart. CGM: continuous glucose monitoring. IGM: intermittent glucose monitoring.

Forty eight VLBW newborns were enrolled in this prospective study, 25 in the CGM-group and 23 in the IGM-group (Fig. 1) between November 2008 and November 2009. The patients’ characteristics are described in Table 1. The two groups were similar at inclusion, except for maternal-fetal infection that was present only in the CGM-group (n = 6). Gestational age was <32 weeks for most of the patients except two in the IGM-group and one in the CGM-group. At inclusion, caloric intakes from birth were similar in both groups ((IGM-group 25.8 ±3.7 vs CGM-group 26.4 ± 4.6 kcal/kg/d, not significant)). Continuous glucose monitoring was started between 6 and 27 hours of life, with a median duration of recording of 71.8 [43.5–87.7] hours, with no difference for both groups. Four monitorings failed, two in each group, due to technical problems during the insertion of the sensor, including angulations of the sensor in three cases and minor local bleeding in one case. One patient was excluded directly after the inclusion because of a branchiooculofacial syndrome. Finally, 43 monitorings could be analyzed, 21 and 22 in the IGM- and CGM-groups respectively. A post-hoc analysis confirmed that glucose levels estimated by the CGMS were highly correlated to the corresponding capillary blood glucose values (linear regression, p<0.0001, r² = 0.96) and the difference between the two techniques was ≤ 10 mg/dl for 93% of the values.

**Table 1:** Characteristics of the 48 very low birth weight newborns with intermittent (IGM) or continuous glucose monitoring (CGM).

	**IGM**	**CGM**
N	23	25
Gestational age (weeks)	29.6 [24.1–34.7]	30.1 [24.4–37]
Birth weight (g)	1014 [579–1485]	1000 [620–1485]
Small for gestational age	10 (43%)	9 (36%)
Gender male	13 (57%)	13 (52%)
Outborn	1 (4%)	4 (16%)
Maternal diabetes mellitus	1 (4%)	1 (4%)
Apgar score <5 at 5 min	2 (9%)	1 (4%)
Umbilical arterial pH <7.1	1 (4%)	1 (4%)
Severe respiratory distress syndrome	6 (26%)	5 (20%)
Maternal-fetal infection	0	6 (24%)*
Age at inclusion (h)	17.9 [6.4–27.3]	15.8 [7.0–25.0]
Duration of glucose monitoring (h)	71.8 [43.5–71.9]	71.9 [47.7–87.7]
Glucose monitoring failure	2 (9%)	2 (8%)

Results are expressed as number of patients (%) or median [min-max].

*p<0.05

### Hypoglycemia Detection: Capillary Blood Versus Blind Continuous Glucose Monitoring (IGM Group)

**Figure 2:**
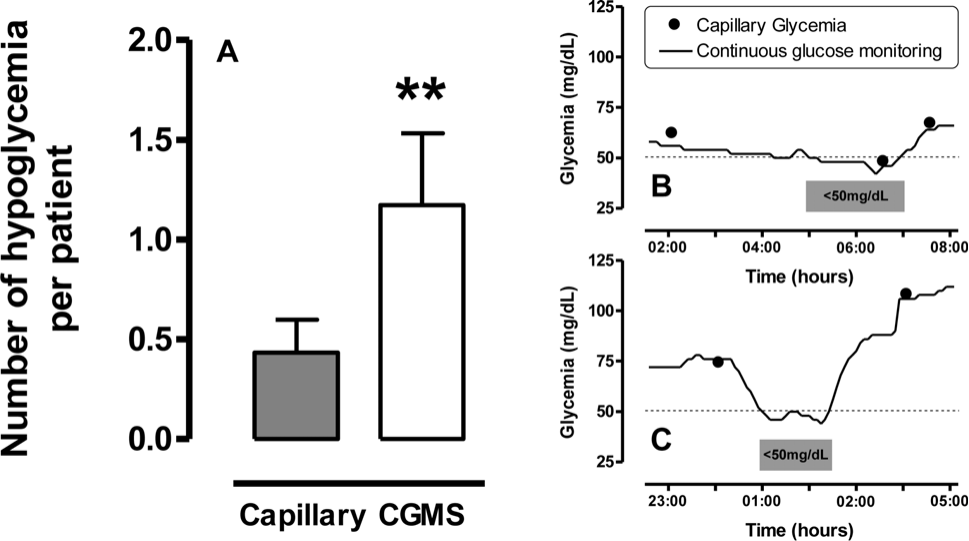
Number of hypoglycemic episodes per patient in the IGM-group and examples of hypoglycemic episodes. Number of hypoglycemic episodes per patient detected by capillary blood glucose testing or continuous glucose monitoring in the IGM-group (A) and examples of hypoglycemic episodes (B and C). In 21 patients from the IGM-group, glucose level was monitored by capillary blood glucose testing performed every 4 hours and by blind continuous glucose monitoring system (CGMS, Guardian Clinical, Medtronic) that was analyzed retrospectively. Hypoglycemia was defined by a glucose level ≤50mg/dL. Number of hypoglycemia episodes per patient are expressed as mean ± SE; **p<0.01. In the first example (B), blind continuous glucose monitoring detected a hypoglycemic episode 100 min before capillary blood glucose testing. In the second example (C), hypoglycemic episode (90 min of duration) was detected solely by continuous glucose monitoring.

In the IGM group, capillary blood glucose testing performed every 4 hours detected 10 hypoglycemic episodes ≤50 mg/dl in 7 patients. Blind-CGMS, which was analyzed retrospectively, detected a significantly higher number of hypoglycemia episodes (n = 27, Fig. 2A) than capillary blood glucose testing (1.2±0.4 vs 0.4±0.2 episode/patient, p<0.01). The 10 previously described episodes were detected prematurely in 8 cases, 59 ± 19 min before capillary blood glucose testing. The 17 other hypoglycemia episodes were not identified by capillary blood glucose testing in a total of 11 patients. Some examples of monitoring are shown in Fig. 2B and 2C.

### Hypoglycemia Management: Benefits of Real-Time Continuous Glucose Monitoring (IGM- Versus CGM-Group)

**Figure 3:**
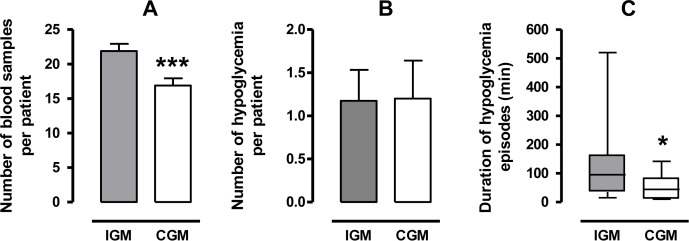
Comparison between IGM- and CGM-group: number of heel pricks, number and duration of hypoglycemic episodes per patient. Number of heel pricks per patient for capillary blood glucose testing (A), number (B) and duration (C) of hypoglycemic episodes per patient. Results are expressed as mean ± SE (A and B) or as median, 25% and 75% percentiles (box) and extreme values (whisker) (C) *p<0.05, ***p<0.001.

**Figure 4:**
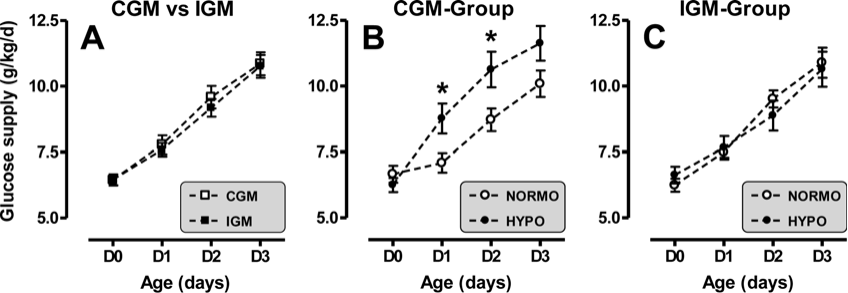
Daily carbohydrate supplies in IGM- and CGM-group. Results, expressed as mean ± SE, represent daily carbohydrate supplies during the first 4 days of life in IGM versus CGM-group (A), and in patients with (HYPO) versus without (NORMO) hypoglycemia in each group (B and C); *p<0.05.

In the CGM-group, the use of RT-CGMS made it possible to significantly decrease the number of blood samples for capillary blood glucose testing by 25% (p<0.001, Fig. 3A). Continuous glucose monitoring identified 30 hypoglycemia episodes in 11 patients, which was similar to the results in the IGM-group (1.2±0.4 episode/patient, Fig. 3B). The characteristics presented in table 1 were not different between patients with or without hypoglycemia (S1 Table, in supplemental appendix). The use of RT-CGMS made it possible to significantly reduce the median duration of the hypoglycemic episodes per patient by 50%, from 95 [15–520] min to 44 [10–140] min (p<0.05, Fig. 3C). A higher number of intravenous dextrose bolus was performed in the CGM-group but the difference was not significant(1.27±0.38 vs 0.64±0.20 bolus/patient, p = 0.16). The mean daily caloric and carbohydrate supplies were similar in each group during the study (Fig. 4A). However, post-hoc analyses demonstrated that the daily carbohydrate supply was significantly higher at day 1 and 2 for patients with hypoglycemia compared with patients without hypoglycemia in the CGM-group (Fig. 4B). This difference was not statistically significant in the IGM-group (Fig. 4C).

## Discussion

Continuous glucose monitoring detected a significantly higher number of hypoglycemic episodes than repeated capillary blood glucose testing, as demonstrated in the IGM-group with the use of a blind-CGMS. Indeed, glucose concentrations ≤50mg/dl were detected in 22 neonates (51%) from both groups during their first days of life. This higher rate of hypoglycemia detection was also reported in the previous studies using CGMS, and confirms that hypoglycemia is more frequent, and most of the time asymptomatic, in VLBW babies, than reported in the past [19, 21, 22]. Furthermore, the use of CGMS in a real-time mode provided new insight into the management of hypoglycemia and enabled them to be treated earlier and to reduce their duration. More specifically, RT-CGMS made it possible to identify a sub-group of patients with hypoglycemia during their first days of life, in accordance with wide variation in the use of energy and glucose requirements in VLBW neonates [23, 24]. In these patients, the glucose supply was increased prematurely, i.e. at the time of hypoglycemia, with the result that episodes were reduced. The risk of hypoglycemia was not predictable, considering that these patients with higher intakes of glucose during their first days of life could not be differentiated from other VLBW neonates by their demographic or perinatal characteristics. Thus, the use of RT-CGMS enabled the identification of these neonates and provided the opportunity for an individual management of the glucose supply, closest to their needs. Concerning the imbalance for risk factors of hypoglycemia between the two groups (see Table 1), most of these factors (maternal-fetal infection and outborn patients) had a higher frequency in the CGM group, confirming the beneficial effect of monitoring glycemia with RT-CGMS to reduce the duration of hypoglycemic episodes. More in details, all the patients with maternal-fetal infection (n = 6) were in the CGM-group. After exclusion of these patients, the number of hypoglycemic episodes per patient remained not different between the 2 groups (IGM 1.3±0.4 vs CGM 1.4±0.6, p = 0.78) and the mean duration of these episodes tend to be shorter in the CGM group but the difference did not reach significance (IGM 95 [15–520] min vs CGM 50 [10–140] min, p = 0.14). The same has been applied for outborn patients: exclusion of these patients (1 with hypoglycemia in the IGM-group, and 4, 2 of 4 with hypoglycemia, in the CGM-group) let to no difference in the number of hypoglycemic episodes per patient (p = 0.83) and a difference of duration almost statistically significant (IGM 107 [15–520] min vs CGM 50 [10–140] min, p = 0.053).

Reducing the duration of low glucose concentrations may be beneficial for neonates, notably from a neurological point of view. However, the definition and significance of hypoglycemia remains one of the most confused and controversial issues in contemporary neonatology [14]. It is classically admitted that a blood glucose level higher than 50 mg/dl may be safe. Retrospective studies performed in premature and VLBW patients tried to correlate the characteristics of the hypoglycemia episodes with the risk of neurological sequelae. Firstly, Koh *et al.* outline the relationship between a blood glucose concentration <50 mg/dl and abnormal evoked potentials, whereas clinical symptoms are most of the time absent [15]. At the same time, a large detailed multicenter study of 661 preterm infants suggests that repeated episodes of moderate hypoglycemia (<50 mg/dl) have serious neurodevelopmental consequences with reduced psychomotor developmental scores at 18 months of age [7]. Psychomotor delay and microcephaly persist up to the age of 5 years [8]. Based on these studies, we decided to use 50 mg/dl as the level for hypoglycemia in our work. Thus, the use of RT-CGMS, by detecting earlier and more hypoglycemia episodes, enabledus to reduce their duration and possibly to improve the neurological outcome.

We also demonstrated that CGMS was easy and safe to use in VLBW newborns. Continuous interstitial glucose monitoring was initially developed for the management of diabetes mellitus, resulting in the improvement of metabolic control [18]. The use of this device in VLBW newborns was initially reported in two studies performed in respectively 16 and 38 premature babies weighing from 547 to 1400 g, but using the older CGMS version which allowed only a retrospective analysis of the data stored in the device [20, 21]. More recently, the usefulness and reliability of RT-GCMS was demonstrated in babies born at ≥ 32 weeks gestation who were at risk of hypoglycemia [19]. Our study focused on VLBW babies, a population with a high risk of hypoglycemia, and confirmed the feasibility and safety of the RT-CGMS in these babies treated in an intensive care unit. We experienced four monitoring failures, principally at the beginning of the study and related to minor technical problems during the insertion of the sensor. This failure rate will be probably improved in the future by the increasing experience of the nurses using this device regularly, and by the reduction of the electrode size (Enlite Sensor^®^, Medtronic^®^, Nothridge, USA, 69% reduction in size compared to previous sensor)[25]. Only one patient developed a local infection and recovered without sequelae after the ablation of the sensor. Even if the present study was not designed to assess the precision of glucose measurement, we demonstrated that interstitial glucose levels estimated by RT-CGMS were highly correlated to the corresponding capillary blood glucose values. Similarly, previous studies have clearly demonstrated the closer correlation between blood and interstitial glucose concentrations [19, 20, 21, 26]. In addition, the use of RT-CGMS significantly decreased the number of blood samples for capillary blood glucose testing, potentially reducing the distress and the blood spoliation associated with repeated blood sampling [27].

The use of RT-CGMS, which reduces the duration of hypoglycemia episodes be they moderate or asymptomatic, may improve neurological outcome in a population of VLBW neonates. Additionally, the development of a new algorithm to anticipate hypoglycemia may potentially prevent hypoglycemia episodes and reduce their frequency [28]. This device could also be used in regard of hyperglycemia risk, for VLBW newborns, in hyperinsulinism or in neonates with a risk of glucidic intolerance, to help to manage insulin infusion.

No financial support was received from Medtronic by any of the coauthors or their respective institutions or belonging professional associations.

## Supporting Information

S1 CONSORT Checklist CONSORT Checklist. (DOC)

S1 Protocol Trial Protocol. Research protocol for the trial.
(DOC)

S1 Table Characteristics of the 43 very low birth weight newborns with or without hypoglycaemia. (TIF)
