# Supplementary material for: Real-Time Continuous Glucose Monitoring Reduces the Duration of Hypoglycemia Episodes: A Randomized Trial in Very Low Birth Weight Neonates
Source: PLoS One. 2015 Jan 15;10(1):e0116255. doi: 10.1371/journal.pone.0116255 (PMC4295867; doi:10.1371/journal.pone.0116255)
Supplement: S1 Protocol — Research protocol for the trial. (DOC) [file pone.0116255.s002.doc]

**Real-time continuous glucose monitoring reduces duration of hypoglycemia episodes: a randomized trial in very low birth weight neonates**

PURPOSES OF THE STUDY:

(1) Scientific justification:

The repeated, even moderate hypoglycemia, are a risk factor of bad long-term neurological development for premature newborn children.

(2) Hypothesis:

The screening of hypoglycemias as well as their mangment in newborn children of low birth weight is based on the rigorous monitoring of the glycemia.

(3) Purposes of the study:

Primary outcome:

Compare both strategies of monitoring of the glycemia (intermittent capillary sample and continuous glucose monitoring) on the screening of the number of hypoglycemias.


Secondary outcomes:

x Prevention of hypoglycemias with the real time continuous monitoring system:

decrease of the number of hypoglycemia between both groups between the continuous and blind

decrease of their duration

x Decrease of the capillary blood samples

x comparison of the glucidic and caloric intakes

· MATERIAL(EQUIPMENT) AND METHOD:

(1) Patients:

- Criteria of inclusion:

- Newborn children hospitalized in the Department of Neonatology of the University Hospital of Tours,
- Of a birth weight < 1500 g,
- before 24 hours of life

- Criteria of not inclusion:

- Contraindication in the installation of the sensor ( severe cutaneous damages)
- Transfer towards another hospital center scheduled(programmed) within 4 days,
- Severe Genetic Pathology syndrome malformatif, endocrine pathology

(2) Inclusion and randomization

Written consent delivered to the parents with collection of consent before the 24th hour of life.

Randomization of the newborn children at least of 1500g in 24 first hours of life in two groups by drawing lots:
- group 1: capillary blood samples and continous glucose monitoring with delayed interpretation
- group 2 : real time continous glucose monitoring

Stratification in 2 under groups according to the birth weight:
- under group A: newborn children of lower or more equal born weight in 1000g
- under group B: newborn children of lower or more equal born weight in 1500g and strictly upper in 1000g

Randomization and stratification according to the list of randomization supplied by the Laboratory of Biostatistics, Epidemiology, Medical Computing

(3) Monitoring of the glycemia and managment of the hypoglycemias:

Monitoring of the glycemias and the coverage(care) of the hypoglycemias according to protocols used in the service(department) (cf annex).

(4) Assessment criteria:

- Number and duration of hypoglycemias 0,5g / l in the group RT-CGMS and the number of hypoglycemias detected by the capillary glycemias (group IGM) and by the holter blind
- Number of capillary glycemias
- Caloric and glucidic intakes from J0 to J3
- Unwanted Effects in connection with the glycemic holter

(5) Projected duration of the study: 1 year
