# Supplementary figures and images for: Real-Time Continuous Glucose Monitoring Reduces the Duration of Hypoglycemia Episodes: A Randomized Trial in Very Low Birth Weight Neonates
Source: PLoS One. 2015 Jan 15;10(1):e0116255. doi: 10.1371/journal.pone.0116255 (PMC4295867; doi:10.1371/journal.pone.0116255)

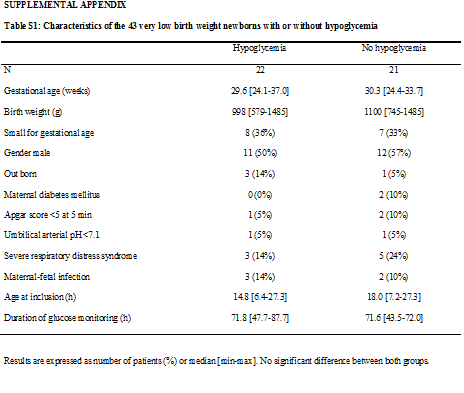

Supplement: S1 Table — (TIF) [file pone.0116255.s003.tif]
